# Exosomes: potential targets for the diagnosis and treatment of neuropsychiatric disorders

**DOI:** 10.1186/s12967-024-04893-6

**Published:** 2024-01-29

**Authors:** Haorao Li, Yanling Yuan, Qinglian Xie, Zaiquan Dong

**Affiliations:** 1 Department of Pharmacy, West China Hospital, Sichuan University, Chengdu, 610041 People’s Republic of China; 2 Department of Outpatient, West China Hospital, Sichuan University, Chengdu, 610041 People’s Republic of China; 3 Department of Psychiatry and National Clinical Research Center for Geriatrics, West China Hospital, Sichuan University, Chengdu, 610041 People’s Republic of China; 4 Mental Health Center, West China Hospital, Sichuan University, Chengdu, 610041 People’s Republic of China

**Keywords:** Neuropsychiatric disorders, Exosomes, Diagnostic marker

## Abstract

The field of neuropsychiatry is considered a middle ground between neurological and psychiatric disorders, thereby bridging the conventional boundaries between matter and mind, consciousness, and function. Neuropsychiatry aims to evaluate and treat cognitive, behavioral, and emotional disorders in individuals with neurological conditions. However, the pathophysiology of these disorders is not yet fully understood, and objective biological indicators for these conditions are currently lacking. Treatment options are also limited due to the blood–brain barrier, which results in poor treatment effects. Additionally, many drugs, particularly antipsychotic drugs, have adverse reactions, which make them difficult to tolerate for patients. As a result, patients often abandon treatment owing to these adverse reactions. Since the discovery of exosomes in 1983, they have been extensively studied in various diseases owing to their potential as nanocellulators for information exchange between cells. Because exosomes can freely travel between the center and periphery, brain-derived exosomes can reflect the state of the brain, which has considerable advantages in diagnosis and treatment. In addition, administration of engineered exosomes can improve therapeutic efficacy, allow lesion targeting, ensure drug stability, and prevent systemic adverse effects. Therefore, this article reviews the source and biological function of exosomes, relationship between exosomes and the blood–brain barrier, relationship between exosomes and the pathological mechanism of neuropsychiatric disorders, exosomes in the diagnosis and treatment of neuropsychiatric disorders, and application of engineered exosomes in neuropsychiatric disorders.

## Introduction

The nervous system is the most intricate and crucial regulatory system in the human body. Neuropsychiatric disorders generally comprise compromised nervous system structure and function [1]. Neuropsychiatry is based on the idea that the body and mind are mutually reinforcing [2]. Several challenges in the diagnosis and treatment of neuropsychiatric disorders include the lack of objective biomarkers, blood–brain barrier (BBB) blockage, drug tolerance, and poor drug targeting [3]. For example, no specific diagnostic indicators for diseases such as schizophrenia (SCZ) exist. Moreover, even with drug therapy, approximately one-third of the patients with SCZ still develop drug resistance [4]. Therefore, elucidating the underlying pathophysiology of neuropsychiatric disorders is important to identify disease-specific indicators and develop novel diagnostic and treatment approaches.

Exosomes, which are nanoscale vesicle structures produced by cells, have several advantages in terms of drug delivery. First, exosomes are derived from an individual’s own cells and have low toxicity [5]. Second, exosomes have low immunogenicity since they are distributed throughout the body, including tissues, blood, urine, and breast milk. Third, exosomes allow for targeting since they contain specific proteins that can act on the corresponding cells [6]. Finally, exosomes can circumvent the P-glycoprotein drug efflux pathway, minimizing drug resistance [7]. Therefore, the significance of exosomes in the occurrence, development, and treatment of various diseases has attracted more and more attention.

Through the biological characteristics of exosomes, they also have certain advantages in the nervous system. Studies have found that exosomes released by healthy individuals and patients carry different biological molecules, such as proteins or RNA, which can be used as a basis for early diagnosis of diseases. In addition, the response to drug treatment can be dynamically monitored, and the recovery of the disease can be judged under the condition of medication [8–10]. In addition, exosomes derived from different cells can reduce neuroinflammation, promote neovascularization, induce neurogenesis, and reduce the apoptotic loss of nerve cells through various mechanisms, which have specific effects on various neuropsychiatric diseases [11–16]. For example, neuron-derived exosomes promote the recovery of functional behavior by inhibiting the activation of M1 microglia and A1 astrocytes [17]. M2 microglia-derived exosomes attenuated ischemic brain injury and promoted neuronal survival via exosomal miR-124 and its downstream target USP14 [18]. Mesenchymal stem cell-derived exosomes (MSC-Exo) can promote angiogenesis and axon growth, regulate inflammation and immunity, inhibit apoptosis, maintain the integrity of blood spinal cord barrier, and play a role in the repair of spinal cord injury [19]. In addition, the discovery of other cell-derived exosomes also has potential therapeutic effects. Exosomes derived from blood Schwann cells can increase the expression of Toll-like receptor 2 (TLR2) in astrocytes through the NF-κB/PI3K signaling pathway, reduce the deposition of chondroitin sulfate proteoglycans (CSPGs), and promote the recovery of injured spinal cord [20]. Lipopolysaccharide (LPS) -stimulated exosomes derived from macrophage RAW264.7 cell line (LPS-Ex) can protect against cerebral ischemia and promote neuroprotection and functional recovery in ischemic stroke by tilting the functional polarity of microglia from M1 to anti-inflammatory M2 phenotype [21]. Herein we aim to discuss the source and biological function of exosomes, relationship between exosomes and the blood–brain barrier (BBB), relationship between exosomes and the pathological mechanism of neuropsychiatric disorders, exosomes in the diagnosis and treatment of neuropsychiatric disorders, and application of engineered exosomes in neuropsychiatric disorders.

## Source of exosomes

**Fig. 1:**
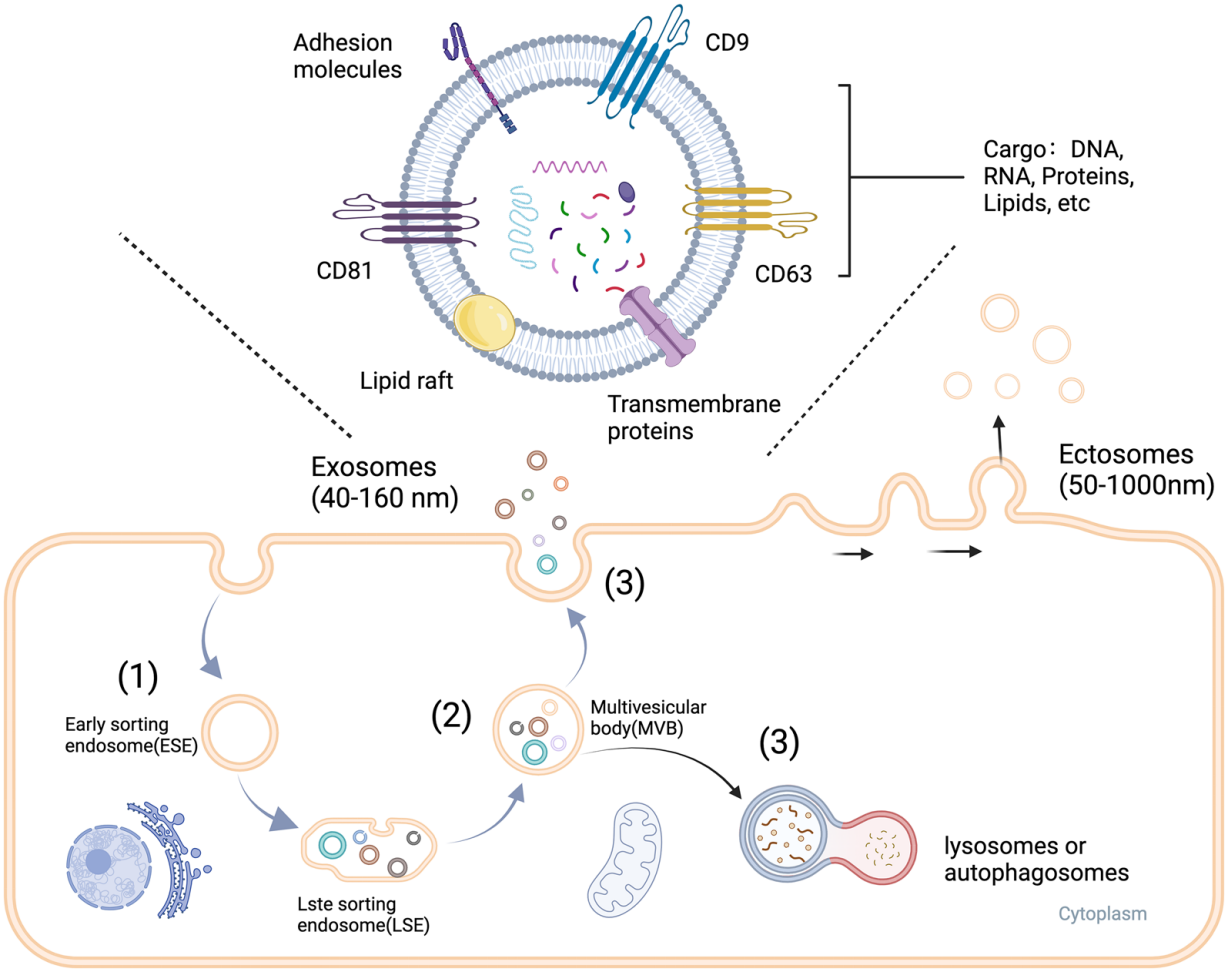
The process of exosome synthesis. EVs contain a lipid bilayer membrane and two subgroups: ectosomes and exosomes. Ectosomes (diameter: 50 nm to 1 m) are vesicles that pinch off the plasma membrane surface by outward budding. Exosomes are endosome-derived EVs ranging in size from 40 to 160 nm. The formation of exosomes occurs in three steps. (1) The first stage is endocytosis of the cytoplasmic membrane to form early sorting endosomes (ESE). At this stage, material on the cell membrane or around the cell is encapsulated into the ESE. (2) In the second stage, the ESE progressively evolves to the LSE. Furthermore, endosomal limiting membrane endocytosis results in the formation of multivesicular bodies (MVBs) containing several intraluminal vesicles (ILVs). (3) The third step involves the destruction of a portion of the MVB by lysosomes or autophagosomes and the fusing of the remaining MVB with the cell's plasma membrane to release the exosomes

All cells produce and release extracellular vesicles (EVs), which can be detected in body fluids such as blood, cerebrospinal fluid (CSF), urine, and breast milk, as well as in various tissues [5, 22]. Furthermore, EVs can be produced by human gut bacteria, which perform several functions in microbial communities by transmitting proteins and genetic material from the bacterium to the host [23, 24]. EVs possess a lipid bilayer membrane and have two subgroups: ectosomes and exosomes. Ectosomes (50 nm to 1 μm in diameter) are vesicles that pinch off the plasma membrane surface via outward budding. Exosomes are endosome-derived EVs ranging from 40 to 160 nm in size [5]. Exosomes were initially thought to be cell fragments that helped cells eliminate waste. However, they have been recently shown to act as bridges between cells and can be used as biomarkers. There are three stages in the generation of exosomes, as shown in Fig. 1. The first stage is endocytosis of the cytoplasmic membrane, which carries the material on the membrane and around the cell to form the early sorting endosome. In the second stage, the early sorting endosome gradually progresses to the late sorting endosome. Furthermore, the endosomal limiting membrane endocytosis forms multivesicular bodies (MVB) containing several intraluminal vesicles (ILVs). The third stage involves lysosome- or autophagosome-mediated degradation of a portion of the MVB and fusion of the remaining MVB with the cell's plasma membrane to release the exosomes [25]. MVB formation is crucial for exosome biogenesis, with membrane budding and ILV generation driving various mechanisms, including the endosomal sorting complex required for transport (ESCRT)-dependent and ESCRT-independent pathways [26]. Classical ESCRT-dependent pathways involve ESCRT-0, -I, -II, -III subcomplexes, and ATPase VPS4, whereas non-canonical pathways use HD-PTP or Alix for specific cargo recognition. Lipid rafts play crucial roles in ESCRT-independent ILV formation [26].

Exosomes contain transmembrane proteins, membrane proteins with lipid anchors, membrane proteins with peripheral associations, and soluble proteins of the exosome lumen, as well as tetraspanins, adhesion molecules, enzymes, scaffolds, and complex glycans. However, a single exosome cannot contain all of these molecules [27]. A previous study identified 22 proteins that were consistently enriched in exosomes that could be potential biomarkers for exosomes. Among them, 15 were intraluminal proteins, six were membrane proteins, and one was an extracellular matrix protein probably connected to the exosome surface. Moreover, syntenin-1 was consistently found to be the most abundant protein in the exosomes [28]. Other reported biomarkers for exosomes include tetraspanins, particularly CD9, CD63, and CD81 [5]. Another study, however, showed that CD63, but not CD9 and CD81, was an exosome-specific marker. Accordingly, EVs expressing CD63 and either or both of the other tetraspanins may be endosome-derived exosomes, whereas those only expressing CD9 or CD81, but not CD63, may be ectosomes [29]. Exosomes are highly heterogeneous and are influenced by various variables, including the substances they contain, their size, their function in recipient cells, and their origin. Exosome changes may be driven by the source donor cells and environment-induced changes in gene expression. A multiplexed analysis of EV approach (MASEV) has been developed to examine the EVs of thousands of individuals through five cycles of multichannel fluorescence staining for 15 EV indicators, which has allowed examination of the biology and heterogeneity of EVs. With technological advances, the diagnostic utility of this approach can be further improved [30]. Therefore, it is important to elucidate the heterogeneity of exosomes to facilitate the study of diseases.

## Biological function of exosomes

Exosomes were first identified as substances that help cells eliminate waste to promote cell survival and maintain cell homeostasis [31]. Exosomes can facilitate the removal of toxic nuclear DNA from the cytoplasm, inhibiting the release of exosomes, which result in cytoplasmic accumulation of nuclear DNA that triggers the cytoplasmic DNA sensing mechanisms. This triggers a natural immune reaction that activates a DNA damage response mediated by reactive oxygen species (ROS), which results in senile-like cell cycle retardation or apoptosis [32].

The biological function and heterogeneity of exosomes are determined by the state and environment of their donor cells. There has been increasing interest in the composition of exosomes, as well as the phenotypic and molecular changes in exosome-induced receptor cells [5]. Some studies have suggested that the dominant imprinting of parent cells determines the composition and function of exosomes, leading to the proposal of the “exosome parent imprinting” theory. Moreover, the microenvironment regulates the composition and function of exosomes [33]. The therapeutic efficacy of exosomes may be influenced by the age of the donor cell and the oxygen level in the culture [34]. Certain circumstances and stimuli can alter the number and characteristics of astrocyte-derived EVs, which influences the course of Alzheimer's disease (AD) [35]. Furthermore, exosomes derived from umbilical cord mesenchymal stem cells (UMSCs) may prevent myocardial fibrosis and restore cardiac function, with beta-2-microglobulin-deficient UMSC-derived exosomes showing relatively better performance. Therefore, controlling exosome imprinting may enhance the positive effects of exosomes [36].

**Fig. 2:**
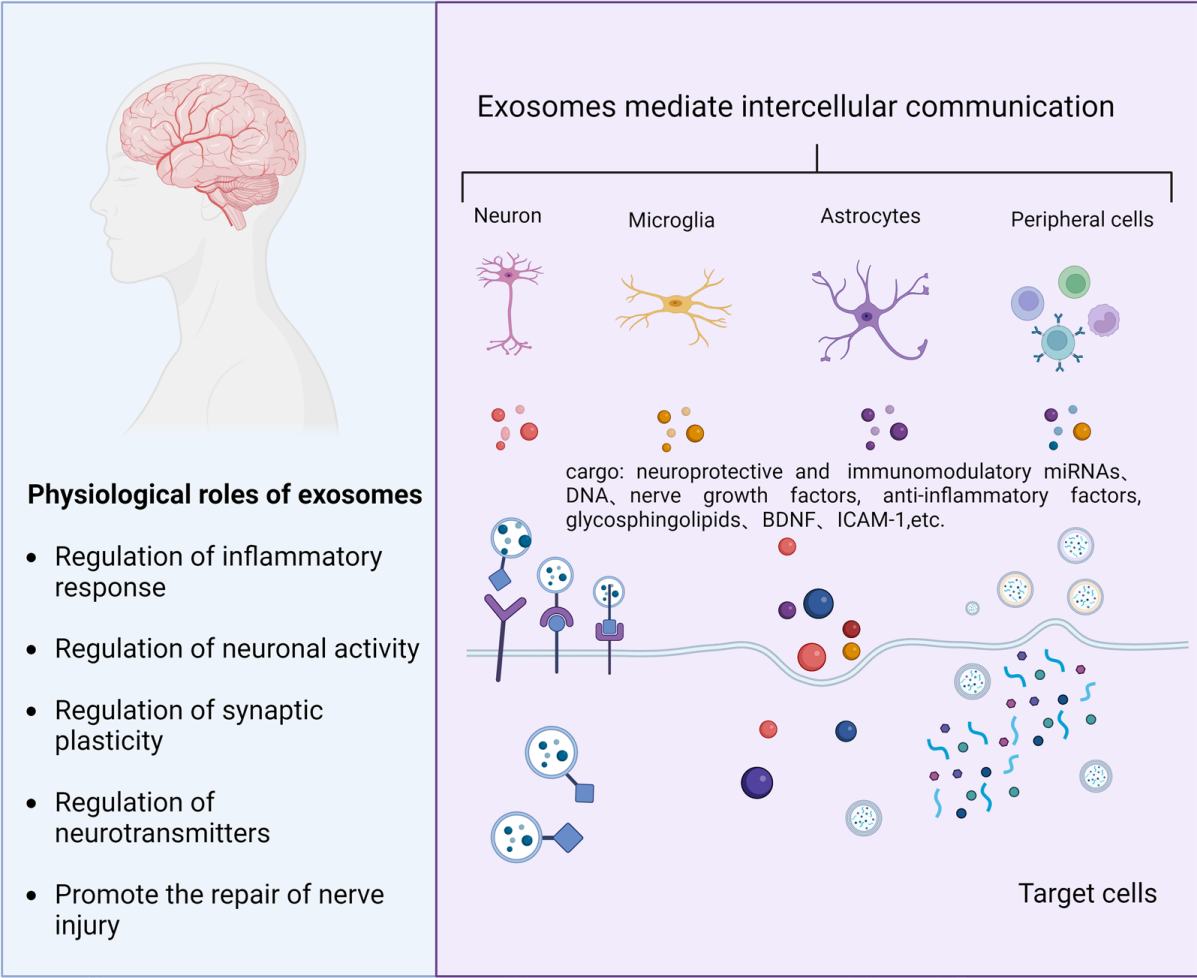
Physiological roles of exosomes. There are abundant substances in exosomes that promote neurodevelopment, including mirnas, etc. These substances interact with target cells, participate in signal transduction and regulate gene expression, thereby regulating inflammatory response and oxidative stress, regulating neuronal activity, regulating neurotransmitter balance, and promoting nerve injury repair. For the study of neurodevelopmental processes, an in-depth understanding of these substances in exosomes will help reveal the fine regulatory network of intercellular communication mechanisms

Taken together, exosomes produced under different conditions have unique compositions and structural characteristics that influence their biological functions, as shown in Fig. 2. including their targeting, biodistribution, biocompatibility, compartmentalization, permeability, and biodegradability [37]. Numerous biomolecules are transported to exert local paracrine or distal systemic effects through the intercellular communication of exosomes [38, 39]. In summary, exosomes are essential in the overall functionality of the body.

## Relationship between exosomes and the blood–brain barrier

The blood–brain barrier (BBB) is composed of complex and dynamic neurovascular units. It controls the exchange of substances between the blood and CNS and provides a relatively safe and stable environment for the brain. However, it impedes the entry of therapeutic agents into the brain. Only 0.1% of therapeutic antibodies administered intravenously reach the brain, resulting in significantly increased circulating concentrations or prolonged administration to reach their target therapeutic concentrations, thereby increasing the risk of systemic toxicity [40]. These disadvantages may be circumvented through the use of exosomes, as they can freely cross the BBB, target the lesion site, and safely return to peripheral circulation. The subsequent section discusses the interaction between exosomes and the BBB observed using imaging technology, as well as the possible mechanism through which exosomes cross the BBB.

Gold nanoparticles have been widely used in therapeutic procedures since they offer versatility in treatment, imaging, and surface modification. Synthetic gold nanoparticles have been paired with the special capabilities of exosome-derived membranes to allow targeted distribution to brain cells [41]. After intranasal administration of GNP-labeled MSC-derived exosomes, computed tomography (CT) imaging revealed increased exosomal accumulation in the brain and long-term presence in the lesion area for up to 24 h [42]. This approach has been used to track the migration and homing characteristics of exosomes derived from bone marrow stromal cells (BMSCs) in neurological diseases. The results further confirmed that their specific and long-term accumulation is dependent on the specific membrane protein composition of MSC-exos. In healthy brains, MSC-exos are eliminated, whereas they accumulate in pathological brain conditions. This indicates the role of chemotaxis-related ligands in exosomes and the exons' innate immunological response to inflammation in MSC-exo homing. Inflammation triggers exosome migration despite neurons in diseased areas being the target cells. Taken together, in vivo neuroimaging using MSC-exos can be used to diagnose neuronal deficits and facilitate the targeted administration of therapeutic agents [43]. Exosomes produced by neutrophils have also been found to be inflammatory chemotactics [44]. Other studies have also found that brain inflammation can enhance the function of the naïve macrophage (Mϕ) exosomes across the BBB. This may be related to the up-regulation of intercellular adhesion molecule-1 (ICAM-1) which is common in inflammation. ICAM-1 can promote the uptake of Mϕ exosomes by the BBB. Brain inflammation often occurs in diseases of the central system. Therefore, some inflammation-related central diseases enhance the delivery of exosomes to the brain, which may benefit the delivery of exosome therapeutic proteins to treat central system diseases [45]. Additionally, radionuclide single photon emission CT and positron emission tomography imaging of radiolabeled exosomes can provide accurate clues regarding their systemic distribution, targeting, and degree of nonspecific tissue uptake. Thus, exosomes can provide practical information regarding brain delivery and therapy. We will further discuss the utility of radionuclide-based exosomes [46].

Exosomes have been studied for their biological significance, as well as potential utility as disease biomarkers and medication carriers. These prospective technological applications necessitate the careful characterization of exosomal BBB permeability and their lipid bilayer composition. A team of researchers created a 3D static BBB system, based on current liposome systems and supplementary LC–MS/MS and ^31^P nuclear magnetic resonance approach, to analyze pure human plasma-derived exosome-like vesicles [47]. Their results demonstrated that exosome-like vesicles have similar BBB permeability as liposomes and can accumulate in endothelial cells. Moreover, the plasma-derived exosome-like vesicles lacked phosphatidylserine (PS) and had a high abundance of lyso species, including phosphatidylcholine, phosphatidylinositol, and phosphoethanolamine [48]. The underlying mechanism could involve the interaction between the bases of fat-soluble molecules and negatively charged phospholipid groups, as well as the increased affinity for the BBB, which allows them to cross the BBB and enter the periphery for cellular communication without removal or degradation [49, 50]. Further, exosomes derived from neural stem cells may act as biological nanocarriers for efficient passage across the BBB. Nanomedicines targeting heparan sulfate proteoglycan may display an increased affinity for brain endothelial cells and subsequent transcytosis across the BBB [51]. Exosomes from primary brain tumors have demonstrated increased expression of Lipocalin-2 (LCN2) in bEnd.3 brain microvascular endothelial cells. Additionally, exosomes have shown improved membrane permeability in bEnd.3 cells in an LCN2-dependent manner. Studies using tandem mass tag quantitative proteomics and bioinformatics have demonstrated that exosomes increase LCN2 expression via the JAK-STAT3 pathway rather than transferring it. This demonstrates how glioma-derived exosomes alter the BBB to allow the passage of nanocapsules via LCN2 [52].

## Relationship between exosomes and the pathological mechanism of neuropsychiatric disorders

### Exosomes and neurodegenerative disease

Neurodegenerative diseases are caused by the loss of neurons and/or their myelin sheath, with dysfunction occurring over time [53]. Neurodegenerative diseases can be divided into chronic neurodegenerative diseases (including includes Alzheimer's disease [AD], Parkinson's disease [PD], and Huntington's disease [HD]) and acute neurodegenerative diseases (including cerebral ischemia, brain injury, and epilepsy).

Abnormal protein conformation is a common pathological feature of chronic neurodegenerative diseases, including amyloidosis, tauopathies, prion diseases, α-synucleinopathies, and mutant huntingtin protein (mHTT) [53]. A large body of literature has reported that extracellular vesicles and their groups are involved in the occurrence of pathogenic proteins. For example, exosomes can participate in the synthesis of misfolded proteins in the brain and promote the development of diseases. Previous studies have found that β-cleavage occurs in early endosomes with subsequent delivery of Aβ to multivesicular bodies (MVBs) in HeLa and N2a cells. Subsequently, a small fraction of the Aβ peptide can be secreted from the cell in combination with exosomes. Exosomal proteins were observed to accumulate in plaques in the brains of AD patients [54]. Under pathological conditions, exosomes can also mediate the dysregulation of amyloid precursor protein (APP) to recruit other extracellular proteins to form senile plaques [55]. Exosomes can also promote the aggregation of toxic proteins.

Extracellular vesicles derived from the cerebrospinal fluid (CSF) of patients with PD have been found to promote α-synuclein aggregation in healthy cells [56]. In addition, it can inhibit the efflux of α-syn in neurons, thereby enabling the pathological accumulation and aggregation of α-syn. Exosomes can also participate in the diffusion of toxic misfolded proteins in the central nervous system [57]. Recent studies have shown that tau is not just an intra-neuronal protein but can also be released into the brain parenchyma through extracellular vesicles, spreading between neurons. Extracellular tau can also be taken up by microglia and astrocytes, degraded by AELN, or proliferated by exosomes [58]. In addition to the ability of exosomes to transduce toxic proteins, genetic components associated with risk genes can also influence the dispersion and pathology of toxic proteins through exosome-related mechanisms. Expression of TREM2 enriched in microglia was associated with an increased risk of sporadic AD by genome-wide association studies (GWAS). A study has demonstrated that Trem2 is a repressor of pathological tau spreading and suggested that Trem2 deficiency can aggravate pathological tau distributing through microglial exosomes [59]. However, when the body is in a pathological state, exosomes may accelerate the progression of the disease. For example, microglia, as macrophages in the brain tissue, regulate the microenvironment of the brain. Some studies showed that after α-synuclein stimulation of microglia in pathological conditions, nucleotide-binding and oligomerization domain-like receptor family pyrin domain containing 3 (NLRP3) inflammasome is activated, which promotes the production and secretion of microglia exosomes and the exosomal transport of α-synuclein between neurons. Exosomes carrying α-synuclein disseminate and accumulate between neurons, leading to degeneration and death of dopamine neurons. The released α-synuclein again stimulates microglia and promotes the aforementioned process, leading to further degeneration and loss of dopaminergic neurons [60]. Exosomal α-synuclein also interacts with microglial toll-like receptor 2 (TLR2), leading to excessive phagocytosis of α-synuclein and activation of microglia, leading to further propagation and spread of α-synuclein pathology [61].

Although accumulation of misfolded proteins is the most common pathological feature of chronic neurodegenerative diseases, neuroinflammation, oxidative stress, abnormalities of the ubiquitin–proteasome and autophagy/lysosome systems, apoptosis, and mitochondrial dysfunction may also participate in the pathological process of neurodegenerative diseases [53]. Exosomes are also involved in these processes. Inflammation is initially a defense mechanism that promotes tissue repair and debris removal. However, chronic inflammation can inhibit cell regeneration and adversely affect the body. The data have shown that EVs can sense and transmit information about peripheral inflammatory states to the central nervous system (CNS), which transmits pro-inflammatory information to recipient brain cells [62]. Researchers have found that plasma EVs from spinal cord injury (SCI) animals boosted reactive astrocyte gene expression and pro- and anti-inflammatory gene expression [63]. The mechanism may be that EV containing pro-inflammatory cargo is released from the spinal cord injury site and delivers the cargo to the brain, affecting the central nervous system [64]. In the central nervous system, microglia are important cells involved in neuroinflammation. Microglia acquire many activation states in response to different local environmental stimuli, ranging from protective to detrimental phenotypes [65]. In addition, miRNA has been found to enter extracellular vesicles through a cellular sorting mechanism, which means that cells can selectively sort miRNAs into EVs and secrete them to nearby or distant targets.

Dysregulation of EV-miRNA has been identified in several disease states, revealing a potential role for their sorting in disease pathogenesis [66]. Studies have shown the influence of various isoforms of non-coding RNAs on inflammatory responses. Exosomes of non-coding RNAs involved in inflammation may be involved in disease development [67]. For example, exosomes can transport miR-21-5P from neurons to microglia, resulting in M1 polarization of microglia and excessive release of pro-inflammatory cytokines, which aggravates neurological impairment [65]. In addition, it has been found that small non-coding RNAs (miRNAs) are more stable in exosomes than in free form. They exert long-lasting effects on the expression of disease-related genes [68]. Oxidative stress involves the aggregation of free radicals produced by the body through inflammation and mitochondrial dysfunction; moreover, it is a common pathological characteristic of nervous system diseases [69]. High oxidative stress levels have been reported in the brains of patients with neurodegenerative diseases [70, 71]. During AD development, pathological tau proteins are distributed by exosomes released by microglia [72]. Insoluble aggregates of tau proteins can induce ROS production in a calcium-dependent manner by activating NADPH oxidase, leading to neuronal damage [73].

**Fig. 3:**
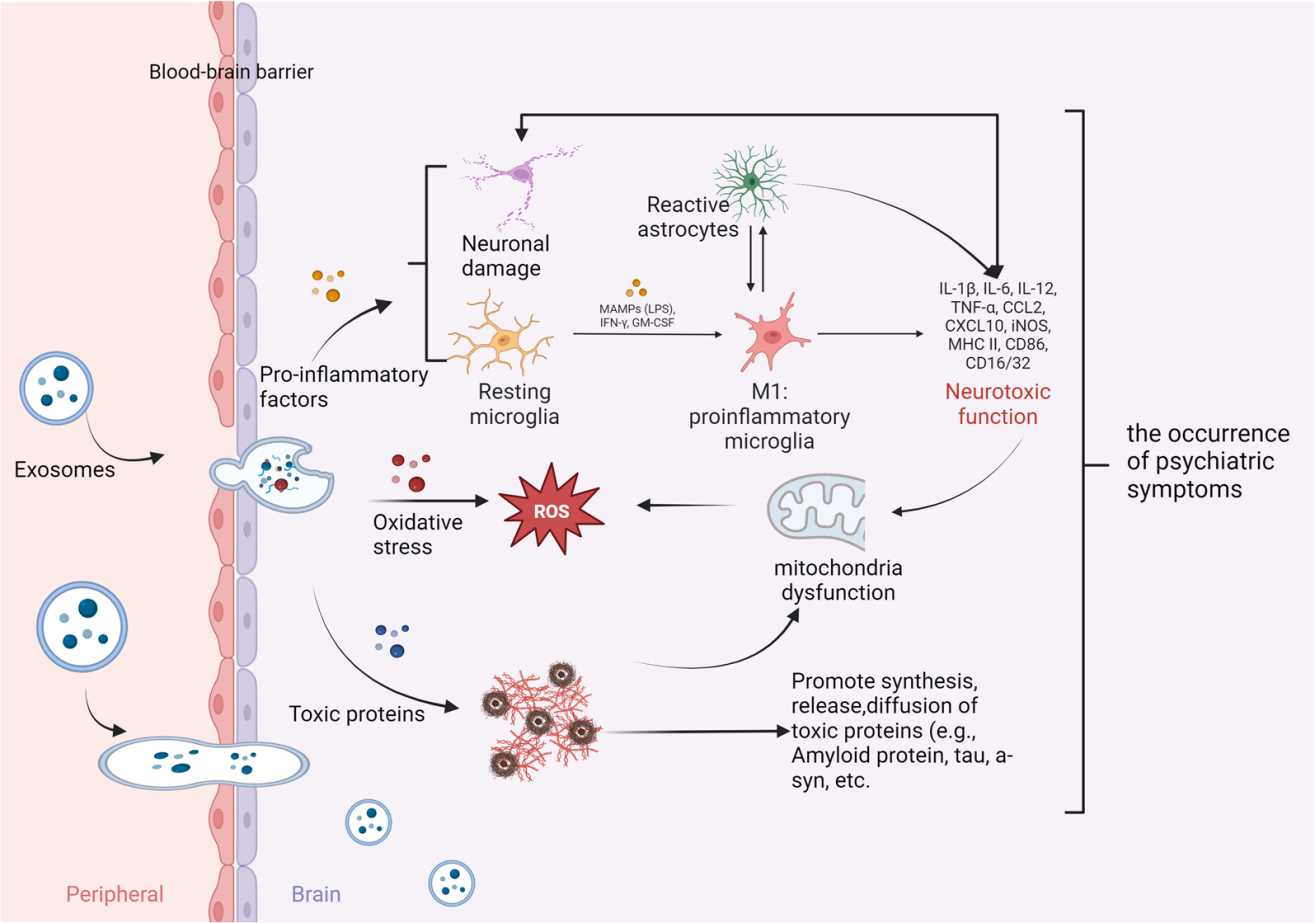
Exosomes are involved in the development of diseases. Exosomes promote the development of neuropsychiatric diseases by spreading toxic proteins, triggering inflammatory responses and oxidative stress, affecting synaptic function and damaging cell metabolism. An in-depth understanding of the mechanism of action of exosomes in neuropsychiatric disorders will help to develop new therapeutic strategies and drug targets, and provide patients with more effective treatment options

Exosomes are also involved in the pathological processes of abnormalities of ubiquitin–proteasome and autophagy/lysosome systems, apoptosis, and mitochondrial dysfunction. The autophagy-lysosome pathway (ALP) maintains the intracellular homeostasis of the cytosolic protein synuclein alpha (SNCA)/alpha-synuclein, which is connected to age-related neurodegenerative synucleinopathies. ALP was found to play an essential role in regulating not only SNCA release but also the protein profile of EVs. This work shows that ALP inhibition increases SNCA in neuronal EVs, different ALP components exist in EVs, and cerebrospinal fluid (CSF) EVs transmit SNCA from cell to cell in vivo [74]. Enhanced autophagosome degradation contributes to the neuroprotective effect on cerebral ischemia–reperfusion injury (CIRI) [75]. The study found that by inhibiting the conversion of autophagosomes to autolysosomes by activating the RIP1/RIP3 pathway, CIRI activated Drp1 and sped up the creation of autophagosomes via p62. However, undegraded autophagosomes were released extracellularly in the form of exosomes, which sparked inflammatory cascades that further harmed mitochondria, produced an excessive amount of reactive oxygen species (ROS), blocked the degradation of autophagosomes, and set up a vicious cycle [76]. The loss of neurons in the brain can lead to abnormal neural network function, which can trigger severe neurological diseases. Polarized microglia play an important role in neurodegenerative diseases and may induce neuronal apoptosis. Studies have found that this may be related to extracellular vesicles secreted by polarized microglia [77]. Mitochondrial dysfunction can trigger inflammation. When mitochondrial malfunction outweighs the neurons' ability to effectively carry out mitochondrial breakdown by autophagy, mtDAMP may promote neuroinflammation. Additionally, in the presence of mitochondrial stress, functioning cells secrete much more mitochondrial DNA and proteins than usual, perhaps in an effort to limit the production of uncontrolled mtDAMPs. However, it is probable that under stressed circumstances, EVs, which have been linked to the activation of the inflammasome, exert a pro-inflammatory impact on the surrounding cells via the EV-associated mtDAMPs. Thus, neuroinflammation and mitochondrial dyshomeostasis may work together to start a vicious cycle that leads to neuronal death [78]. To sum up, the pathophysiological process of each disease is more complex, which will cause the disorder of the internal environment and cause each other to form a vicious circle, as shown in Fig. 3.

### Exosomes and non-neurodegenerative disease

Non-neurodegenerative diseases are relative to neurodegenerative diseases, including depression, bipolar disorder, schizophrenia, and other mental disorders. The pathogenesis of psychiatric disorders is based on various hypotheses, and there is growing evidence for the role of exosomes in brain diseases. Chronic unpredictable mild stress (CUMS) can induce depressive behavior and the expression imbalance of BDNF/TrkB-related synapse-associated proteins and also change the serum exosome profile. Differentially expressed miRNA was also found to potentially target genes involved in key neuronal functions in the brain [79]. The research team found that injecting blood exosomes from patients with depression into the tail vein of normal mice resulted in the development of depressive-like behavior [80]. Inflammation is a common cause of mental disorders. In response to different local environmental stimuli, microglia acquire many activation states, ranging from protective to detrimental phenotypes. For example, exosomes can transport inflammation-related nucleotides from neurons to microglia, resulting in M1 polarization of microglia and excessive release of pro-inflammatory cytokines, which aggravates neurological impairment [81]. Some researchers transplanted serum exosomes from SCZ patients into mice and caused abnormal behavior in mice. In the brains of SCZ exosome recipient mice, methyloxymethanol-treated rats, and SCZ patients, comparative bioinformatics analysis identified shared and distinct differentially expressed genes (DEGs) and enriched molecular pathways. This finding is consistent with altered prefrontal lobe-to-hippocampus functional coherence in SCZ patients. A substantial number of SCZ-relevant DEGs in exosome-recipient mice were targets of differentially expressed (DE) exosomal miRNAs in SCZ patients. The researchers discovered 20 hub genes for SCZ risk genes, including BDNF and NRG1, which were found to be DE miRNA targets in SCZ patients [82]. In summary, external stress may lead to changes in the exosome spectrum of the body and cause the body to lose balance. Simultaneously, exosomes can also transmit information, and a cascade of reactions occur, including transmitting information from the periphery to the central system, leading to the occurrence of psychiatric symptoms, as shown in Fig. 3.

## Exosomes in the diagnosis and treatment of neuropsychiatric disorders

### Neurodegenerative diseases

#### Potential diagnostic function

*Exosomes can be used as liquid biopsies* The diagnosis of the disease is a comprehensive judgment of the patient, which needs to be determined by combining the patient's symptoms and some relevant examinations. Exosomal material generated in the brain can provide important clues regarding the brain, especially under pathological conditions, and can be obtained from various sources, including CSF, urine, whole blood, plasma, and serum [83]. Some studies have verified the concordance between cerebrospinal fluid and blood exosomal biomarkers, and confirmed that exosomal Aβ42, T-tau, and P-T181-tau have the same diagnostic ability as cerebrospinal fluid for AD and amnestic mild cognitive impairment (aMCI) [84]. In clinical practice and research, positron emission tomography (PET) and cerebrospinal fluid (CSF) are expensive and invasive procedures, which hinder further diagnosis of the disease. However, blood measurements are more advantageous in laboratory tests because of their convenient collection, less trauma, and low price. Because exosomes can freely travel between the central and peripheral areas, they are an ideal carrier of biomarkers for disease screening. Animal studies have demonstrated higher peripheral levels of Aβ and tau proteins in neuron-derived exosomes, as well as C1q in astrocyte-derived exosomes, among AD animals than among healthy controls. They mimic the pathological features of AD (senile plaques and hyperphosphorylation of tau) and indicate the potential use of exosomes as liquid biopsies [85]. Moreover, a study reported higher Aβ1-42 and P-S396-tau levels in plasma exosomes of patients with AD than those in matched healthy controls [86]. Additionally, an investigation of large clinical samples and two chronic epilepsy animal models revealed an increase in the levels of two serum exosomal proteins, namely, thrombospondin-1 and coagulation factor IX, which could be potential diagnostic biomarkers for epilepsy [87]. circRNAs in exosomes have been shown to have a long half-life and structural stability [88]. All the above illustrate the reliability of exosomes in blood detection. Technological advancements have increased the capacity to separate and evaluate bulk exosomes in plasma and biofluids. Rapid centrifugation has been used to obtain exosomes from diseased and normal samples. Analysis of the between-group substances in the resulting exosome-derived substances allows the identification of specific genes or proteins, which can be used to inform diagnosis and prognosis [8, 89, 90].

*Exosomes can be used for the early detection of disease and monitoring disease progression* The pathological phenotype of neurodegenerative disease may take years to manifest; therefore, biomarkers are needed for early disease detection. Blood neuro-exosomal synaptic proteins such as GAP43, SNAP25, neurogranin, and synaptotagmin 1 can be used as reliable biomarkers for predicting AD 5–7 years before cognitive decline [91]. For example, the diagnosis of Parkinson's disease is still based on the detection of motor symptoms in clinical practice, but motor symptoms only appear late in the neurodegenerative process, ideally in the prodromal phase of motor symptoms, which is crucial for early treatment of the disease. The detection of pathological a-syn as a neuropathological marker of Parkinson's disease has become the focus of attention. The researchers confirmed the concept that pathologic soluble a-syn, detected in plasma-derived neuronal extracellular vesicles, could serve as a biomarker to distinguish PD patients from healthy controls. The presence of a pathologic soluble a-syn conformation was further confirmed by amplification and ultrastructural analysis of the formed aggregates. Instead of focusing on quantitative levels in body fluids or tissues, detection of pathological neuronal a-syn conformation was targeted in this study [89]. Recently, another research team found that AD measurements of L1EV-synuclein in combination with specific prodromal markers, such as olfactory or cognitive deficit, possible or definite rapid eye movement sleep behavior disorder (RBD), or the *glucocerebrosidase* (GBA1) gene status, could be used to help substratify individuals with the highest risk of developing PD and related Lewy body diseases [92]. To facilitate early diagnosis of neurodegenerative illnesses, another study developed a screening approach that can be used in combination with other recognized subjective evaluation and imaging techniques. Based on the unique surface markers of neuron-derived exosomes, they can be quantified through fluorescence nanoparticle tracking analysis [93].

*Exosomes can predict the disease severity.* Interactions between the presynaptic proteins neuronal pentraxin 2 (NPTX2) and neurexin 2α (NRXN2α) and their respective postsynaptic functional partners, GluA4-containing glutamate (AMPA4) receptor and neuroligin 1 (NLGN1), increase excitatory synaptic activity in the hippocampus and cerebral cortex. NDE levels of all proteins except NPTX2 were considerably lower in a preclinical period, 6–11 years before the onset of dementia, and levels of all proteins fell significantly with the development of dementia. Reductions in neuron-derived exosomes (NDE) levels of these particular excitatory synaptic proteins may thus be indicative of the amount of cognitive loss and of the severity of AD development [94]. The most critical therapy for acute ischemic stroke is to recanalize the occluded artery within a specific time frame in order to save the ischemic penumbral tissue (PEN). The existence of penumbra, however, might fluctuate between hours and days due to the significant heterogeneity of acute ischemic stroke (AIS). The presence of penumbra is extremely useful to a neurologist in selecting whether to undergo reperfusion treatment. CircOGDH was found to be derived from the circRNA of ketoglutarate dehydrogenase, and its expression was significantly upregulated in the brain penumbra of mice with focal ischemia and in the plasma of AIS patients. CircOGDH plays a crucial role in the regulation of neuronal apoptosis. Neuron-derived CircOGDH in the penumbra is enriched in exosomes transported to the peripheral blood during ischemia and is a promising biomarker to predict the presence of the penumbra in AIS patients [95].

*Biomarkers in exosomes can be used for the differential diagnosis of certain diseases* Multiple system atrophy (MSA) and Parkinson’s disease (PD), which often have overlapping symptoms, can be distinguished based on the abnormal accumulation of intracellular α-syn aggregates. Specifically, in oligodendrocytes, α-syn mostly form glial cytoplasmic inclusions in MSA; contrastingly, in PD, α-syn aggregate as intraneuronal Lewy bodies and Lewy neurites. Accordingly, the ratio of α-syn concentrations in putative oligodendroglial and neuronal exosomes is considered a sensitive biomarker for differentiating between PD and MSA [96]. Anti-N-methyl-d-aspartic receptor (anti-NMDAR) encephalitis is a common type of encephalitis with similar clinical characteristics as viral encephalitis. Moreover, there is a lack specific laboratory and brain MRI findings and for a comprehensive differential diagnosis. A study reported a significant increase and decrease in serum exosome miR-140-5P and serum C3 levels, respectively, in patients with anti-NMDAR encephalitis. Therefore, serum exosome miR-140-5P and serum C3 levels can be used to distinguish between anti-NMDAR encephalitis and viral encephalitis [97].

In summary, exosomes as diagnostic tools for neurodegenerative diseases have the advantages of being non-invasive, allowing for early diagnosis with high specificity and diversity, and permitting for dynamic monitoring and differential diagnosis of diseases. Further research may potentially reveal an increasingly crucial role of exosomes in the diagnosis of neurodegenerative diseases.

#### Potential therapeutic effects

Under normal physiological conditions, exosomes help the body maintain homeostasis (Fig. 2). When disease occurs, in order to maintain the normal physiological level of the body, exosomes will also play a positive role (Table 1). For example, it has been found that neuronal exosomes are rich in glycosphingolipids and can capture Aβ. Infusion of neuronal exosomes into the brains of APP transgenic mice reduced the deposition of Aβ and amyloid proteins [98]. Studies have shown that EVs secretion affects the levels of abnormal proteins inside and outside the cell. The primary mechanism is to inhibit autophagosomes, thereby promoting EVs to secrete abnormal proteins (such as Aβ and α-syn), reducing intracellular accumulation and protecting neurons [99, 100]. Repeated mild traumatic brain injury (rmTBI) is thought to be a significant contributor to the development of long-term neurodegenerative diseases like Alzheimer's disease, which is characterized by anomalies in the protein-amyloid and cognitive decline. The researchers found that microglial exosomes with increased miR-124-3p (EXO-124) promoted β-amyloid proteolytic breakdown and reduced neurodegeneration in injured neurons [101]. After an ischemic stroke, white matter restoration is critical for cognitive and neurological recovery. M2-type microglia can communicate with oligodendrocyte precursor cells (OPCs) through M2-evs, and miR-23a-5p may promote white matter repair after ischemic stroke by directly targeting Olig3 [102]. Oligodendrocytes are myelin-forming glial cells that encase neuronal axons and ensure pulse transmission. Ischemic stroke affects not only neurons but also oligodendrocytes. EVs produced by regenerative microglia can restore the function of protective microglia/macrophages, restrict their aging in the post-stroke period, and promote the maturation of OPCs near the lesion edge [103]. Since neurons lack strong antioxidant defenses, astrocytes are crucial for neuroprotection against oxidative stress. They deliver energy substrates, antioxidant enzymes, and antioxidant compounds to help the antioxidant system in neurons [104]. For example, astrocyte-derived exosomes (AS-Exos) defend against oxidative stress and neuronal death caused by TBI by triggering Nrf2 signaling [105].The miRNAs in the exosomes produced by activated astrocytes significantly influence the astrocyte-microglia interaction. By blocking the NF-B signaling pathway, miR-873a-5p, one of the critical components of these astrocyte-derived exosomes, reduced microglia-mediated neuroinflammation and alleviated neurological impairments after TBI [106]. In addition, EVs derived from human deciduous teeth stem cells can promote the polarization of anti-inflammatory microglia to TBI [107]. Human mesenchymal stem cell-derived extracellular vesicles can inhibit the chronic activation of the NLRP3-p38/MAPK signaling pathway, significantly reduce the volume of cerebral cortical lesions, improve cognitive function, and have potential neuroprotective effects [108, 109]. In addition, Brain-derived neurotrophic factor (BDNF) has neuroprotective benefits against therapeutic cerebral ischemia–reperfusion (I/R) damage. Exosomes derived from BDNF-overexpressing HEK293 cells can reduce oxidative stress and calcium ions while maintaining a steady mitochondrial membrane potential in I/R-damaged brain cells [110].

**Table 1:** The therapeutic role of exosomes in neuropsychiatric disorders

Disease	Cell-derived EVs	Cargos	Overcome	References
AD	Neuronal exosomes	Glycosphingolipids	Reduced Aβ and amyloid deposition	[98]
SCI	Neuron-derived exosomes	Up-regulation of miR-124-3p	Inhibition of the activation of neurotoxic microglia and astrocytes protects against traumatic spinal cord injury	[17]
Stroke and demyelinating disease	Microglia-derived EVs	Up-regulation of miR-23a-5p	Promote white matter repair	[102]
TBI	Astrocyte-derived exosomes	NA	Reduce oxidative stress and neuronal apoptosis	[105]
TBI	Macrophage derived exosomes-miR-21 (M-Exos-miR-21)	Down-regulation of miR-21	Improve neuroinflammation, Improve blood–brain barrier (BBB) permeability, Reduced neuronal apoptosis, Recovery of neurological function	[112]
rmTBI	Microglial Exosomal	Up-regulation of miR-124-3p	Inhibition of β-amyloid abnormalities, Alleviates Neurodegeneration and Improves Cognitive Outcome	[101]
Brain damage and neurodegenerative diseases	MSC-derived secretome	NA	Inhibition of reactive astrogliosis, Reduce inflammation, Reduced microglial infiltration, Increased BBB integrity	[114]
MDD	Microglial EVs	Up-regulation of miR-666-3p, Up-regulation of miR-7115-3p	alleviate neuroinflammation	[142]
MDD	NK cell-derived exosomes	Up-regulation of miR-207	Reduced release of pro-inflammatory cytokines Relief of depressive symptoms	[143]
SCZ	MSCs-EVs	NA	Decreased glutamate levels in cerebrospinal fluid and improved core-like behaviors and biochemical markers of schizophrenia	[144]

Some drugs and exogenous substances can promote or inhibit the release of exosomes, or change the composition of exosomes, to alleviate the disease or improve symptoms. EVs derived from neural stem cells are produced more abundantly in response to heat shock (HS), and their morphology and cargo are altered to allow improved neuroprotection against oxidative stress and Aβ-induced neurotoxicity [111]. Exosomes-Mir-21 have successfully reduced neuronal apoptosis, ameliorated neuroinflammation, and increased BBB permeability in the brain at low concentrations. Ginsenoside Rg1 is the main pharmacological active substance of ginseng, which has neurotrophic and neuroprotective effects. Studies have found that ginsenoside Rg1 can prevent the release of Exos-miR-21 from peripheral blood to the brain and play a role in reducing cerebrovascular endothelial damage [112]. A study found that EV/exogenous 27 kDa HS protein (HSP27) mixes could improve the lifespan of brain endothelial cells (through EV mitochondria) and maintain their tight junction integrity (via HSP27 effects) [113]. Systemic administration of EP4 antagonist-elicited MSC-EVs to animals with hippocampal injury was found to enhance BBB integrity, reduce reactive astrogliosis, attenuate severe inflammation, and reverse cognitive, learning, and memory impairments [114]. Neural cells can be triggered by secretory substances from GABA-treated intestinal cells (Caco-2) (SH-SY5Y). Moreover, exosomes derived from GABA-treated Caco-2 cells have been shown to activate the SH-SY5Y cells. These findings indicate that exosomes are secreted by GABA-activated intestinal cells and stimulate brain cells, and miRNAs in such exosomes may significantly influence neuronal cell activation [115]. The cyclical release of melatonin from the pineal gland and retina aids in the coordination of circadian rhythms and neuroendocrine activities [116]. Exogenous melatonin analogues (e.g., ramelteon, agomelatine) are also commonly used in psychiatry to treat sleep disorders or depression with sleep disorders. According to the team’s findings, melatonin administration clearly impacted the number of released exosomes. Limiting exosome release would reduce amyloid beta burden and toxicity. The same team demonstrated that melatonin changed the amount of tau transported by exosomes depending on whether it was administered before or after Aβ [117].

Exosome production can be altered by physical stimuli, including exogenous electrical stimulation, magnetic fields, or ultrasound [118, 119]. Recent research has demonstrated that one of the leading causes of AD is an imbalance between Aβ production and clearance. As a result, increasing exosome production might be a treatment for AD. One team demonstrated, for the first time, that exosomes from ultrasound-stimulated HA cells (US-HA-Exo) may have neuroprotective properties to counteract the cytotoxicity caused by oligomeric amyloid in vitro, and when paired with focused ultrasound (FUS)-induced BBB opening, may make it possible to remove amyloid plaques in vivo [120].

In summary, exosomes, as biological tools with potential therapeutic value, provide new ideas and strategies for the treatment of neurodegenerative diseases via regulating their production and release.

### Non-neurodegenerative diseases

#### Potential diagnostic function

*Exosomes are used as liquid biopsies* Most psychiatric diseases rely on subjective diagnosis, and no objective diagnostic markers exist. Identifying substances contained in EVs in peripheral blood can be used as biomarkers for mental disorders [121]. A previous study successfully detected blood biomarkers related to neuron-derived exosomes using a small amount of peripheral blood. Tumor necrosis factor receptor 1, synaptophysin, and interleukin 34 were strongly associated with the severity of depression and/or other sub-symptoms [122]. Among the many biomarkers, exosomal miRAN is a powerful tool for predicting psychiatric disorders [123–126], It can be identified by EVs in peripheral blood, and some studies have found that the content may be higher in the serum of patients [127–129]. Disease severity can also be reflected by miRNA levels in serum extracellular vesicles [130]. Subthreshold depression is a depressive state that does not meet the diagnostic criteria for major depressive disorder; however, subthreshold depression has the risk of progressing to MDD. Some studies have found that miR-17-5p in extracellular vesicles secreted by brain nerve cells is a biomarker of subthreshold depression. Therefore, extracellular vesicles secreted by brain nerve cells can be extracted from blood, which can be used for early detection and intervention of diseases [131]. In addition, protein expression in exosomes can change through miRNA regulation. The study found that SERPINF1 was significantly reduced in exosomes derived from the peripheral blood of patients with depression, which may result from up-regulation of miR-186-5P. Therefore, detecting SERPINF1 in exosomes can be used to predict depression [121]. Mechanism-based biomarkers that identify abnormal brain circuitry are necessary for the early diagnosis and treatment of schizophrenia. These biomarkers will improve patient classification, illness progression tracking, and therapeutic intervention. Oxidative stress induces the up-regulation of miR-137, which leads to the reduction of COX6A2 and mitophagy markers, the accumulation of damaged mitochondria, and further aggravates oxidative stress and parvalbumin interneuron (PVI) damage. All of these processes were saved by the mitochondrial antioxidant MitoQ. Blood exosomal miR-137 increases and COX6A2 reductions in early psychosis patients (EPP) and changes in mitophagy markers imply that both central and peripheral animal model data were mirrored in the patients' blood. A decrease in ASSR gamma oscillations in the EEG was correlated with higher exosomal miR-137 and lower COX6A2 levels. Alterations in plasma exosome levels of miR-137/COX6A2 may serve as a surrogate marker of PVI cortical microcircuit damage since ASSR depends on healthy PVI-related networks [132].

*Exosome-mediated sensitive biomarkers* The research team constructed lncRNA-miRNA-mRNA networks using high-throughput sequencing to identify the expression levels of lncRNA, miRNA, and mRNA in plasma exosomes from healthy (HCs) and first episode schizophrenia (FOS) patients. Correlation analyses were performed between lncRNAs and PANSS score, miRNAs and mRNAs in the network, GO and KEGG pathway, and PPI network of mRNAs in the network. Finally, lncRNA-miRNA-mRNA networks were discovered as sensitive diagnostic indicators, offering fresh perspectives on illness detection [133]. Recently, 25 exosome-derived metabolites were also found to have good to excellent performance in distinguishing SCZ patients from HCs [134]. Similarly, dysregulation of exosome metabolites has also been found in bipolar disorder (BD). The results showed that there were 26 differentially expressed metabolites in serum exosomes, which were enriched in glucose metabolism-related pathways. Among them, 15 exosome metabolites could distinguish BD from MDD and SCZ [135]. In addition, when BD begins with a depressive episode, it often needs to be differentiated from depression in the diagnosis. Serum bacterial-derived extracellular vesicles have been found to be useful in differentiating bipolar disorder from major depressive disorder. The prevotella 2 and Ruminococcaceae UCG-002 genera were identified as potential candidates [136].

In summary, changes in specific nucleic acids, proteins, and other substances related to mental disorders in exosomes can be used for the auxiliary diagnosis of diseases, including early detection and intervention of diseases, judgment of disease severity, tracking of disease progression, and differential diagnosis of diseases. Simultaneously, these exosome changes could aid in the treatment of mental disorders.

#### Potential therapeutic effects

The pathogenesis of mental disorders is complicated by a combination of hereditary and societal variables. The study found that aerobic exercise is a non-pharmacological strategy for treating depression, which may be achieved by regulating inflammation. This may be mediated by altering circulating EV signatures, which are associated with reduced systemic inflammation [137]. The results of another study in AD patients suggest that the cognitive benefits of exercise may be achieved by elevating neuron-derived extracellular vesicle (NDEVS)-mediated neuroprotective factors. In addition, AD patients carrying APOE ε4 are more sensitive to the neuroprotective effects of physical activity [138].

A large number of studies have found that exosomes derived from different cells are rich in neuroprotective and immunomodulatory miRNAs, nerve growth factors, and anti-inflammatory factors, which can reduce neuroinflammation, promote new blood vessel formation, induce neurogenesis, reduce neuronal apoptosis loss, and improve cognition [17, 108, 139, 140]. Microglia are the innate immune effector cells of the central nervous system, which mediate the endogenous immune response to central nervous system injury and disease, thus playing a neuroprotective or neurotoxic role. Studies have found that microglia transfer microglia-enriched microRNA, miR-146a-5p, through exosomes, thereby inhibiting neurogenesis in depression [141]. Astrocytes are the most abundant cells in the central nervous system, and their activation leads to the release of inflammatory cytokines. Glutaminase1(GLS1) is expressed in microglia and participates in inflammation. GLS1 deficiency can enhance the levels of miR-666-3P and miR-7115-3P released by microglia in extracellular vesicles, thereby inhibiting the expression of Serpina3n and inhibiting the activation of astrocytes [142]. In addition, Tril is highly expressed in astrocytes and can activate transcription factors such as NF-κB, thereby inducing the production of pro-inflammatory cytokines. Studies have found a high level of miR-207 in the exosomes of NK cells, which can target Tril to inhibit the NF-kB signaling pathway of astrocytes and alleviate depressive symptoms [143]. Exosomes derived from mesenchymal stem cells (MSC-Exo) are rich in neuroprotection-related substances and have great potential in treating neurocognitive disorders [11]. For example, MSC-derived EVs decreased CSF glutamate levels in phencyclidine-treated mice, which may contribute to the reduced toxicity. This suggests that MSC-EVs ameliorate key biochemical markers and behaviors associated with SCZ and are a potential treatment option for SCZ [144]. Therefore, exosomes can regulate gene expression, neurotransmitter levels, and inflammation to improve neurological dysfunction in psychiatric disorders.

Based on the pathogenesis of mental disorders, it has been found that some drugs in the treatment of mental disorders may be mediated by exosome induction. For example, neuroinflammation has been implicated in the development of bipolar disorder. A study using NEV biomarkers found that infliximab, a tumor necrosis factor-alpha (TNF-α) antagonist, improved depressive symptoms in bipolar disorder patients with a history of childhood abuse. The drug may participate in the TNFR/NF-κB neuroinflammatory pathway in a childhood trauma-dependent manner, affecting clinical responses and brain structural changes [145]. Interferon gamma-stimulated dendritic cell exosomes (IFN-DC-Exos) greatly enhance myelination and reduce oxidative stress, which could mitigate depression symptoms. Moreover, IFN-DC-Exos injections have shown potential in migraine treatment [146]. Antipsychotics affect the exosomal production of a psychosis-altered and glial-enriched miRNA that regulates neuronal gene expression [123]. Global miRNA alterations in plasma EXO from SCZ patients were shown to be linked to neuronal functioning, with clozapine therapy increasing hsa-miR-675-3p expression [147].

In summary, the pathogenesis of psychiatric disorders usually involves information transmission between neurons; exosomes can regulate this process by carrying bioactive substances. Therefore, by changing the characteristics of circulating exosomes, a therapeutic effect can be obtained. However, current research on the human translation data of exosomes in psychiatric disorders remains limited, and most of the research is in the laboratory stage, mainly using methods such as cell culture and animal models. In the future, more clinical trials are needed to verify the role of exosomes in the treatment of psychiatric disorders.

## Application of engineered exosomes neuropsychiatric disorders

### Neurodegenerative diseases

There has been increasing interest in exosomes since they are comparable in size to liposomes and exhibit low immunogenicity. They belong to the class of nanoscale vesicles, and their non-lamellar lipid content allows a favorable curvature and facilitates drug administration [148]. Exosome-mediated drug delivery systems involve loading substances, including nucleic acid proteins, chemicals, and natural drugs, into the inner cavity or membrane of purified exosomes. These drug delivery systems have high BBB permeability and can reduce the adverse effects of medications. Compared with free medications, these systems allow for a superior therapeutic effect and a higher delivery efficiency [149, 150]. These systems involve two main methods: drug loading into exosomes (including co-incubation, electroporation, and ultrasound) and drug introduction into exosome-derived cells [151]. Moreover, genetic engineering or chemical modification can alter the exosomal surface to improve medication targeting, reduce toxicity, and enhance the therapeutic effect [152]. The following subsections discuss some of the benefits of using exosomes for drug delivery:

*Improved targeting:* Because the complexity of the brain hinders the treatment of brain diseases, there is a need for both precise targeted therapeutic agents and safe and effective drug-delivery systems. Exosomes play a therapeutic role and serve as an ideal delivery system. Silibinin (Slb) has been shown to enhance behavior and cognitive function in patients with AD by lowering amyloid-β (Aβ) aggregation and deactivating astrocytes. However, its low ability to target the brain and poor bioavailability limit its broad utilization. Slb was encapsulated in macrophage-derived exosomes (Exo-Slb) to increase its brain-targeting capabilities. Exo-Slb is preferentially associated with Aβ monomers to minimize aggregation and was internalized in astrocytes to regulate their activation and ameliorate astrocyte inflammation-mediated neuronal injury. Finally, Exo-Slb significantly improved cognitive impairments in AD mice [153]. In addition to their natural targeting ability, exosomes can be modified to meet other needs. Several studies have shown that enkephalins may promote neuronal survival; however, they are restricted by the BBB. Given the high expression of transferrin receptors on the BBB, a previous study combined transferrin with enkephalin-loaded exosomes to yield "tar-exo-enkephalin,” which could successfully cross the BBB [154]. Traditional medicine has attracted much attention for several years. Corynoxine-B, a bioactive substance of Rhynchophylline, is effective in treating AD; however, the BBB prevents its absorption. Engineering hippocampal neuron cell-derived exosomes to overexpress Fe65 has created a targeted drug delivery system that delivered Corynoxine-B (Cory-B, an autophagy inducer) to APP overexpressed-neuron cells in the brains of AD mice [155]. The Fe65-EXO-Cory-B promoted autophagy in APP-expressing neuronal cells, which improved cognitive loss and pathology in AD animals [155]. In addition, the cyclo (Arg-Gly-Asp -D-Tyr-Lys) polypeptide [c(RGDyK)] with high affinity to integrin α_v_β_3_ of ischemic cerebrovascular endothelial cells was conjugated to the surface of mesenchymal stromal cells (MSC)-derived exosomes. c(RGDyK)-conjugated exosomes (cRGD-Exo) can target the lesion area of the ischemic brain and enter microglia, neurons, and astrocytes. Curcumin, a natural polyphenol extracted from turmeric, was loaded onto cRGD-Exo. The results showed that cRGD-Exo-cur inhibited the inflammatory response and apoptosis in the lesion area more effectively than curcumin or exosomes alone [156]. Similarly, another group created a recombinant fusion protein containing the arginine-glycine-aspartic acid (RGD)-4C peptide (ACDCRGDCFC) linked to the lactadherin (C1C2) phosphatidylserine (PS)-binding domains, which rapidly self-associates onto the EV membrane. It can significantly improve its targeting and effectively inhibit post-stroke inflammation [157]. In addition, the study group has developed magnetic nanovesicles (MNV) derived from MSC-loaded iron oxide nanoparticles (IONP), which can significantly improve the targeting of ischemic injury and treatment outcomes. IONP can stimulate the expression of therapeutic growth factors in MSC, and MNV contains more therapeutic growth factors. Magnetic navigation improves the ability of MNV to localize to ischemic lesions. It can also promote the anti-inflammatory response, angiogenesis, and anti-apoptosis in the ischemic brain area, thus significantly reducing the infarct volume and improving motor function [158].

*Reducing toxicity* Exosome-containing drugs can reach the designated sites, which circumvents the toxicity of free-circulating drugs. Alzheimer's disease (AD) is characterized by mitochondrial dysfunction as a key pathogenic characteristic. However, present mitophagy inducers' toxicity and poor brain enrichment restrict their potential applicability. The team developed a non-therapeutic platform for AD using nanoscale mesenchymal stem cell-derived extracellular vesicles (MSC-EVs-SHP2) that highly express tyrosine phosphatase-2 (SHP2). In AD mice, MSC-EVs-SHP2 has a high BBB penetration ability and promotes the delivery of SHP2 to the brain. It can significantly induce mitophagy in neuronal cells, reduce mitochondrial injury-mediated apoptosis and NLRP3 inflammasome activation, and ultimately alleviate synaptic loss and cognitive decline [159]. Some studies have taken advantage of the natural brain-targeting ability of blood exosomes and successfully loaded dopamine into them using a saturated solution incubation method. This system showed improved dopamine distribution in the brain, improved treatment effects against PD, and reduced systemic toxicity of dopamine [160].

*Exosomes can protect drug stability*: In vitro and in vivo studies have shown that exosomes can slow the degradation of drugs and increase their stability after encapsulation. Antisense oligonucleotides (ASOs) can reduce the expression of α-syn and have a certain role in treating PD. However, ASOs are difficult to pass through the cell membrane and are prone to rapid degradation by proteases. Exosomes-mediated ASO4 delivery (exo-ASO4) was demonstrated to have high cellular uptake and low toxicity in vivo and in vitro. It can significantly reduce the expression of α-syn and weaken its aggregation. The degeneration of dopaminergic neurons was ameliorated, and the motor function was significantly improved [161]. Similarly, ASOs are a new therapeutic strategy for Huntington's disease (HD), an autosomal dominant neurodegenerative disease. The exosomal recycling system and artificial genetic circuits were utilized to self-assemble and deliver mHTT silencing siRNA to the cortex and striatum [162]. Another study involved curcumin loaded into exosomes that inherited the specific active targeting function of lymphocyte function-related antigen 1 and endothelial intercellular adhesion molecule 1 from their parent cells, which inhibited Tau phosphorylation through AKT/GSK-3β pathway activation. Accordingly, the exosome-loaded curcumin could better prevent neuronal death in vivo and in vitro, as well as alleviate AD symptoms [163]. In addition, in addition to the ability to maintain the stability of drugs, exosomes also have a certain sustained release effect. For example, loading curcumin into exosomes produced by human endometrial stem cells showed sufficient stability and sustained release [164]. ExoCAT obtained using sonication, in addition to showing high loading efficiency and preserving the enzymatic activity of CAT, also showed prolonged and sustained release, with < 40% of the CAT being released over 24 h [165]. Tom40 is a critical mitochondrial membrane protein that can protect neurons from oxidative stress by improving mitochondrial function. The study found that the protein expression significantly decreased in patients with neurodegenerative diseases. However, getting protein to the cell may be tricky. The BBB in particular is a significant barrier to overcome in order to get the protein to the brain. Exosomes can be used as delivery vesicles to package Tom40 across the BBB. Exosome-mediated delivery of Tom40 was found to protect cells from oxidative stress induced by hydrogen peroxide, which may facilitate the treatment of neurodegenerative disorders such as AD and PD [166].

*Nucleic acid delivery* A major challenge for all gene therapy techniques is the distribution of recombinant DNA to target cells. Exosomes have recently been proposed as a natural means of precisely delivering medications and biological materials to target cells [167]. A previous study confirmed that mature miRNAs were packed into exosomes produced by transfected cells when miR-29a and miR-29b expression vectors were used to transfect HEK-293T cells and BMSCs. Subsequently, they investigated the possibility of using modified exosomes to minimize the negative effects of the amyloid-(A) peptide in a rat AD model with spatial learning and memory abnormalities. These abnormalities were abolished by injecting the rats with exosomes containing miR-29 [168]. Another study used reagent transfection to investigate the exosomal loading of exogenous miR-494, which effectively decreased the inflammatory response and neuronal apoptosis in the injured area, as well as upregulated anti-inflammatory factors and miR-494 to exert neuronal protective effects. In addition, exosomal miR-494 helped restore behavioral function and promoted neurofilament rebuilding in SCI mice [169].

### Non-neurodegenerative diseases

Based on the great advantages of exosomes as drug delivery systems, engineered exosomes also have certain potential for studying psychiatric disorders. The traditional treatment for perimenopausal depression is antidepressant combined with estrogen (E2); however, E2 has more side effects. Pituitary adenylate cyclase-activating polypeptide (PACA) is an endogenous neuropeptide, a neuromodulator, neurotrophin, or neurotransmitter involved in various functions in the central nervous system. The research team created a nanogel called hyaluronic acid nanogel (HA NGs) @exosomes that is coated with exosomes, loaded with PACAP and estrogen (E2), and sensitive to reactive oxygen species (ROS). In vitro and in vivo experiments have proven that HA NGs@exosomes efficiently enter cells and cross the BBB. Ovariectomized rats under chronic unexpected mild stress (CUMS) performed better behaviorally after receiving intranasal administration of HA NGs@exosomes, demonstrating that these exosomes had a quick-acting antidepressant effect. The expression of crucial proteins in the PACAP/PAC1 pathway may be regulated by HA NGs@exosomes, which also have considerable anti-inflammatory and antioxidant characteristics and are thought to enhance synaptic plasticity [170]. Examination of circRNAs has provided clues regarding the pathophysiology of MDD involving the interaction between genes and the environment from the perspective of epigenetics [171]. CircDYM is a homologous circRNA found in both humans and mice. Its plasma and hippocampal levels are noticeably downregulated in individuals with MDD and in depression-like animal models. A team recently accomplished targeted delivery of circDYM to the CNS. Specifically, ultracentrifugation was used to separate RVG-circDYM-ev from circDYM-overexpressing HEK 293T cells, followed by transportation of circDYM to the brain in order to treat depression-like behavior induced by persistent unexpected stress [172]. Several chemicals, especially miRNAs with pleiotropic regulation, have been linked to psychiatric diseases. Therefore, further research on exosomes and their contents could yield promising biomarker candidates that may inform therapeutic strategies and help elucidate the genetic networks involved in the mechanisms underlying mental diseases [173]. Although research is still in its preliminary stage, advances in cell culture technology, exosome extraction and purification technology, and engineered exosomes for targeted drug delivery could allow the application of these findings in clinical practice.

## Conclusions

The biological characteristics of exosomes, such as their small size, low toxicity, low immunogenicity, and targeting ability as a drug delivery system, allow for the improved the therapeutic effect of drugs, reduced drug side effects, and improved quality of life of patients. In addition, exosomes can promote the regeneration and repair of nerve tissue, thus advancing the development of regenerative medicine for neuropsychiatric diseases. Consequently, a deepening understanding of the composition of exosomes could help the field of pharmaceutical research and enable the selection and modification of the bioactive molecules of exosomes more precisely, allowing for the precise intervention of diseases. Therefore, exosomes have broad application prospects in neuropsychiatric diseases.

## Data Availability

Not applicable.

## Abbreviations

AD: Alzheimer's disease
BD: Bipolar disorder
BBB: Blood–brain barrier
BDNF: Brain-derived neurotrophic factor
BMSCs: Bone marrow mesenchymal stem cells
CNS: Central nervous system
CT: Computed tomography
ESCRT: Endosomal sorting complex required for transport
EVs: Extracellular vesicles
GBM: Glioblastoma multiforme
I/R: Ischemia/reperfusion
LCN2: Lipocalin-2
MDD: Major depressive disorder
MSC: Mesenchymal stem cell
MSA: Multiple system atrophy
MASEV: Multiplexed analysis of EV approach
MVB: Multivesicular bodies
NADPH: Nicotinamide adenine dinucleotide phosphate
PMCAO: Persistent middle cerebral artery occlusion
ROS: Reactive oxygen species
SCI: Spinal cord injury
TMZ: Temozolomide
TLR: Toll-like receptor
TBI: Traumatic brain injury
UMSCs: Umbilical cord mesenchymal stem cells
